# Report of two patients in whom comparisons of the somatic mutation profile were useful for the diagnosis of metastatic tumors

**DOI:** 10.1186/s40792-022-01566-8

**Published:** 2022-12-02

**Authors:** Kenichiro Furukawa, Keiichi Hatakeyama, Masanori Terashima, Keiichi Fujiya, Yutaka Tanizawa, Etsuro Bando, Takashi Sugino, Kenichi Urakami, Tateaki Naito, Hiroyasu Kagawa, Ken Yamaguchi

**Affiliations:** 1 Division of Gastric Surgery, Shizuoka Cancer Center, 1007 Shimonagakubo, Nagaizumi-Cho, Sunto-Gun, Shizuoka, 411-8777 Japan; 2 Medical Genetics Division, Shizuoka Cancer Center Research Institute, 1007 Shimonagakubo, Nagaizumi-Cho, Sunto-Gun, Shizuoka, 411-8777 Japan; 3 Division of Pathology, Shizuoka Cancer Center, 1007 Shimonagakubo, Nagaizumi-Cho, Sunto-Gun, Shizuoka, 411-8777 Japan; 4 Division of Thoracic Oncology, Shizuoka Cancer Center, 1007 Shimonagakubo, Nagaizumi-Cho, Sunto-Gun, Shizuoka, 411-8777 Japan; 5 Division of Colon and Rectal Surgery, Shizuoka Cancer Center, 1007 Shimonagakubo, Nagaizumi-Cho, Sunto-Gun, Shizuoka, 411-8777 Japan; 6 Shizuoka Cancer Center, 1007 Shimonagakubo, Nagaizumi-Cho, Sunto-Gun, Shizuoka, 411-8777 Japan

**Keywords:** Diagnosis of a metastatic tumor, Somatic mutation comparison, Next-generation sequencing, Gene mutational signature, Panel sequencing

## Abstract

**Background:**

When a patient has multiple tumors in different organs, it is very important to identify whether the tumors are multiple cancers or metastasis from one tumor in order to establish an optimal treatment strategy. However, it is difficult to obtain an accurate diagnosis from conventional diagnostic strategies, including immunohistochemistry. We report two patients with multiple tumors in which a somatic mutation comparison using next-generation sequencing (NGS) was useful for the diagnosis of a metastatic tumor.

**Case presentations:**

Patient 1: A 64-year-old man was diagnosed with gastric and lung cancer. After radical chemoradiotherapy for lung cancer, gastrectomy was planned for gastric cancer. At gastrectomy, the patient underwent a multiple omics analysis for “Project HOPE”. The gene mutational signature of the gastric tumor showed signature 4 of COSMIC mutational signature version 2, which was associated with smoking and has not been found in gastric cancer. To confirm that the gastric tumor was metastasis from lung cancer, we conducted a somatic mutation comparison of the two tumors with 409-gene panel sequencing, which revealed that 28 of 97 mutations in the lung tumor completely matched those of the gastric tumor. Based on these findings, the gastric tumor was diagnosed as metastasis from lung cancer. Patient 2: A 47-year-old woman underwent distal gastrectomy for gastric cancer. A colon tumor was detected 6 years after gastrectomy. The colon lesion was a submucosal tumor-like elevated tumor, and was suspected to be metastasis from gastric cancer. The patient underwent sigmoidectomy, and participated in “Project HOPE”. The possibility of primary colon cancer could not be ruled out, and we conducted a somatic mutation comparison of the two tumors as we did with Patient 1. Panel sequencing revealed 11 mutations in the gastric tumors, 4 of which completely matched those of the colon tumor. The colon tumor was diagnosed as metastasis from gastric cancer.

**Conclusion:**

We reported two patients with multiple tumors in which a somatic mutation comparison using NGS was useful for the diagnosis of a metastatic tumor.

**Supplementary Information:**

The online version contains supplementary material available at 10.1186/s40792-022-01566-8.

## Background

Patients with multiple tumors in different organs are sometimes encountered. In order to establish an optimal treatment strategy, it is very important to identify whether the tumors are multiple cancers or metastasis from one tumor.

In the differential diagnosis for cancer, immunohistochemical staining is sometimes applied; however, it is difficult to obtain complete diagnosis in every patient. The molecular profiles of primary tumors and metastasis have been extensively compared [1–3]. Recently, with the advancement of next-generation sequencing (NGS), several studies have focused on gene alterations between primary cancers and their metastases, and found specific mutations in both the primary tumor and metastasis [4–10]. However, there are no reports demonstrating the clinical usefulness of identifying somatic gene mutations in paired tumors from the same patient.

We herein report two patients in which a somatic mutation comparison using NGS was useful for the diagnosis of patients with multiple tumors. This is the first report to describe patients in whom a mutation comparison contributed to the clinical diagnosis.

## Case presentations

### Patient 1

A 64-year-old man was diagnosed with gastric cancer after complaining of epigastric pain. During a detailed examination of his gastric cancer, a lung tumor was found. He was then referred to our hospital.

He had smoked 20 cigarettes per day from 20 to 64 years of age, and did not have a drinking history. The results of a hematological examination were within the normal ranges. The patient’s carcinoembryonic antigen (CEA) level was elevated to 72.4 ng/mL, whereas his neuron specific enolase, cytokeratin 19 fragment (CA19-9), and pro-gastrin-releasing peptide levels were within the respective normal ranges.

Endoscopically, the gastric lesion was a type 2 tumor of 30 mm in size. Contrast-enhanced computed tomography (CT) showed irregular thickening of the stomach wall, two swollen lymph nodes at the splenic hilum and along the splenic artery, and a solid tumor of 10 mm in size in the upper lobe of the right lung. ^18^F-fluorodeoxyglucose-positron emission tomography (FDG-PET) showed the accumulation of FDG in swollen mediastinal lymph nodes near the main bronchus, as well as the lung tumor, gastric tumor, and swollen lymph nodes in the abdominal cavity. Bronchoscopic lung biopsy and endoscopic ultrasound-guided fine needle aspiration biopsy of the swollen mediastinal lymph node were performed.

**Fig. 1:**
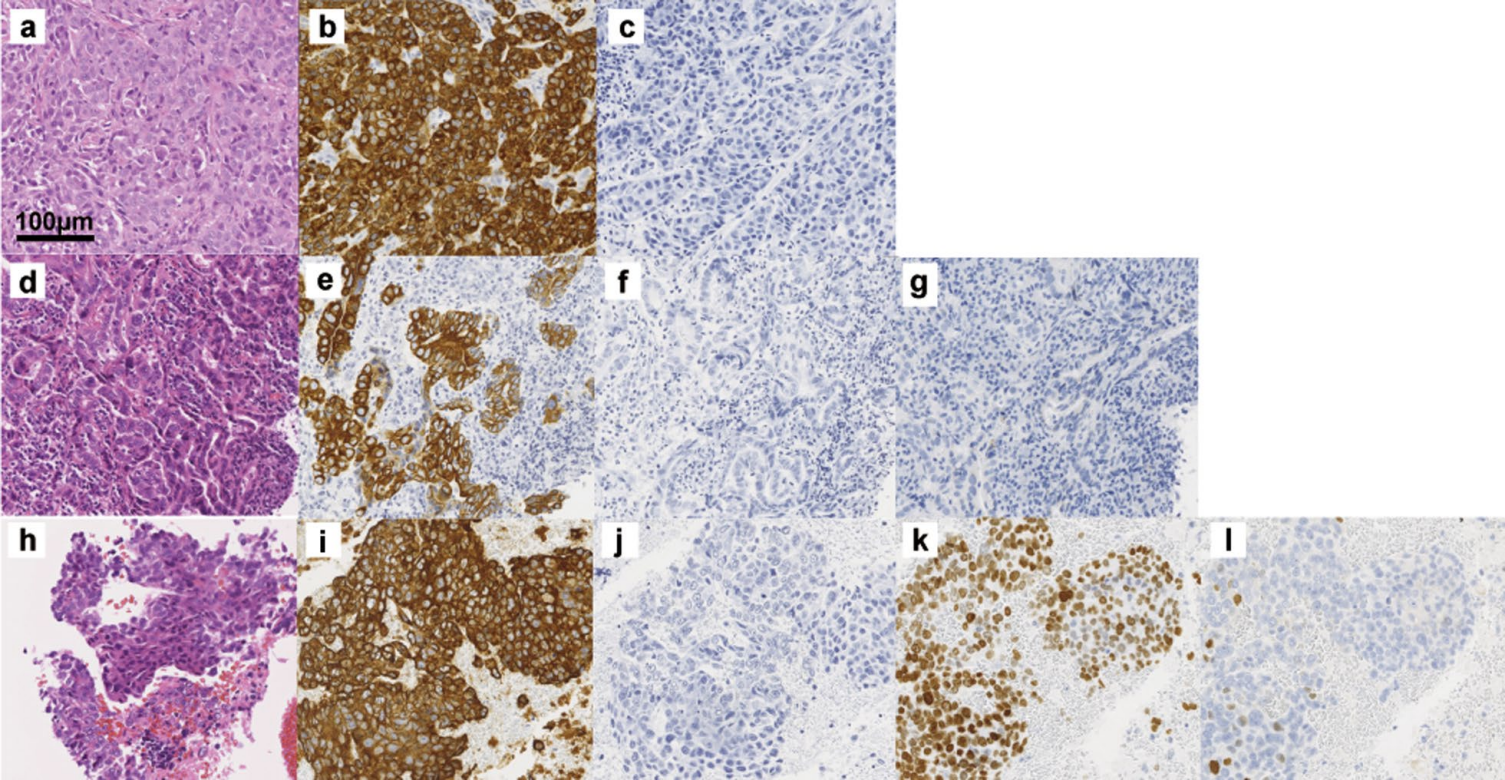
Histopathological examination of biopsy specimens. Specimens from a gastric tumor biopsy (**a**–**c**), lung tumor biopsy (**d**–**g**), and mediastinal lymph node biopsy (**h**–**l**). Histopathological examination of the gastric tumor biopsy specimen showed poorly differentiated adenocarcinoma (hematoxylin–eosin staining) (**a**). The tumor cells of the gastric tumor were positive for cytokeratin (CK)7 (**b**) and negative for CK20 (**c**). Biopsy of the lung tumor showed invasive adenocarcinoma (hematoxylin–eosin staining) (**d**). Part of the tumor was tubular adenocarcinoma with mucus production. The tumor cells of the lung tumor were positive for CK7 (**e**), negative for CK20 (**f**), and negative for TTF-1 (**g**). Biopsy of the mediastinal lymph node showed poorly differentiated adenocarcinoma without a gland duct (hematoxylin–eosin staining) (**h**). The tumor cells of the mediastinal lymph node were positive for CK7 (**i**), negative for CK20 (**j**), positive for TTF-1 (**k**), and focally positive for p40 (**l**)

The histopathological examination of biopsy specimens from the gastric tumor and lung biopsy specimen revealed poorly differentiated adenocarcinoma and invasive adenocarcinoma, respectively (Fig. 1a, d). Mediastinal lymph node biopsy showed poorly differentiated adenocarcinoma (Fig. 1h). Immunohistochemical staining of the gastric tumor, lung tumor, and mediastinal lymph node were performed (Fig. 1b, c, e–g, i–l).

The gastric and lung tumors were diagnosed as discrete primary cancers, because of the different histopathological findings. The clinical stages of the cancers were cStage IIIB (T4a N2 M0) gastric cancer according to the Japanese Classification of Gastric Carcinoma, 3rd English edition [11], and stage IIIB (T1a N3 M0) lung cancer according to the Union International for Cancer Control-TNM, 8th edition.

**Fig. 2:**
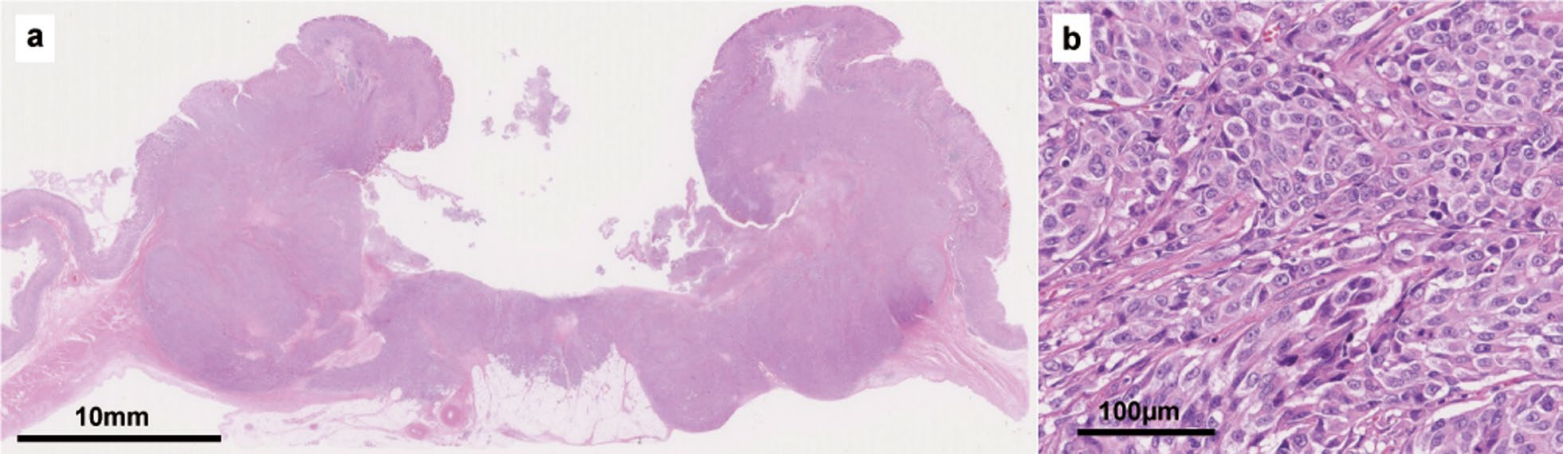
Histopathological findings of the gastric tumor. The ulcerated tumor with raised clear margins was 55 mm in size, and non-cancerous mucosal tissue covered the raised margin of the tumor (**a**). The tumor consisted of solid type poorly differentiated adenocarcinoma (hematoxylin–eosin staining) (**b**)

After a multidisciplinary team conference, curative chemoradiotherapy was indicated for the lung cancer. Four courses of cisplatin plus S-1 and thoracic radiation (60 Gy in 30 daily fractions) were performed. After chemoradiotherapy, the lung tumor shrank and the gastric cancer did not show any obvious change. Hence, gastrectomy was performed. At gastrectomy, the patient participated in the multiple omics analysis for “Project HOPE”, and whole exome sequencing (WES) and gene expression profiling (GEP) were conducted [12, 13]. Macroscopically, the lesion was an ulcerated tumor of 55 mm in size with raised clear margins (Fig. 2a). The raised margin of the tumor was covered by non-cancerous mucosal tissue. Microscopically, the tumor consisted of solid type poorly differentiated adenocarcinoma (Fig. 2b). Chemotherapy showed no therapeutic effect. A histopathological examination revealed pStage IIIB (T4a N2 M0) gastric cancer. The patient underwent adjuvant chemotherapy with S-1; however, CT showed lymph node recurrence in the abdomen six months after gastrectomy. The patients died 11 months after gastrectomy.

**Fig. 3:**
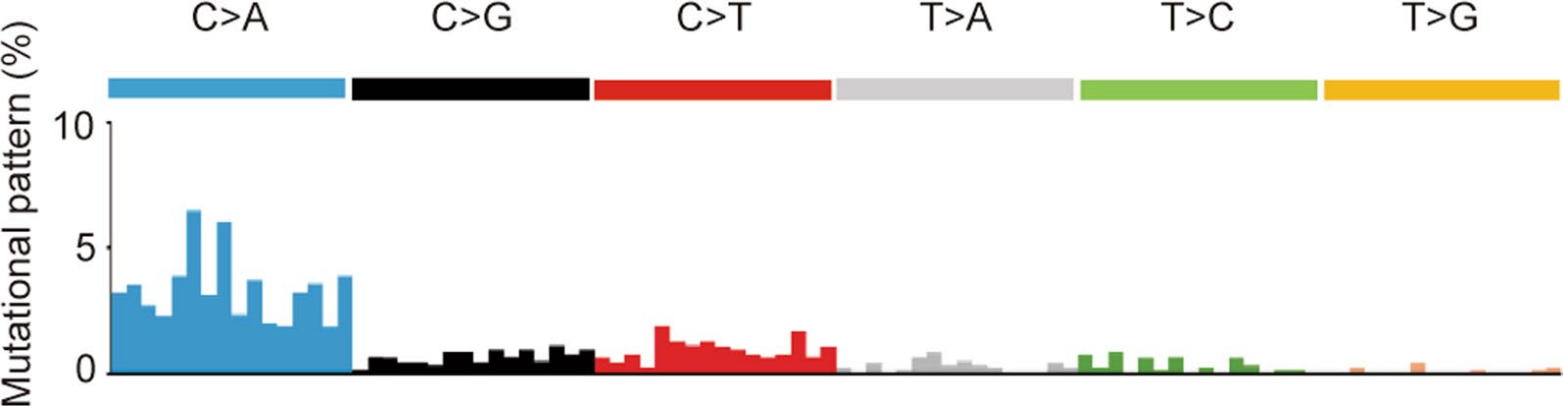
COSMIC mutational signature of the gastric tumor. The signature showed signature 4 (version 2 in COSMIC [https://cancer.sanger.ac.uk/cosmic/signatures_v2]), which was associated with smoking and which has been found in lung cancer, head and neck cancer, and liver cancer. The order of mutation patterns conforms to COSMIC

The gene mutational signature of the gastric tumor showed signature 4 of COSMIC mutational signature (version 2; https://cancer.sanger.ac.uk/cosmic) (Fig. 3). Signature 4 is associated with smoking and has been found in lung cancer, head and neck cancer, and liver cancer [14]. However, there are no reports of primary gastric cancer showing signature 4. We were concerned about the possibility of a metastatic gastric tumor from lung cancer, because the patient had lung cancer and a smoking history. TTF-1 is well-known to be a transcription factor that is highly expressed in thyroid, brain and lung cancer. Thus, additional TTF-1 immunohistostaining of the gastric tumor biopsy and gastrectomy specimens was performed. Both the gastric tumor biopsy and gastrectomy specimens were TTF-1 (+), suggesting that the gastric tumor was metastasis from lung cancer (Additional file 1: Fig. S1a, b). However, TTF-1-positive primary gastric cancer could not be completely ruled out because there are reports describing TTF-1-positive gastric adenocarcinoma [15, 16].

To confirm the origin of the gastric tumor, we conducted a somatic mutation comparison of the two tumors after obtaining permission from the institutional review board. DNA extracted from formalin-fixed paraffin-embedded tissues of the lung biopsy and resected stomach were compared using a custom 409-gene panel sequencing including cancer related genes. Detailed experimental protocols have been previously described [13]. To exclude germline variations, blood-derived WES results were used as a control. Panel sequencing revealed 97 and 87 mutations in the lung and gastric tumors, respectively (Additional file 2: Table S1). Of these mutations, 5 and 7 mutations in the lung and gastric tumors, respectively, were driver mutations including likely-pathogenic mutations (tier 1 and 2 in our previous study) [13]. Of the 97 lung tumor mutations, 28 completely matched (including chromosomal location and pattern of the mutation) the mutations of the gastric tumor. Of these mutations, two were driver mutations. The gastric tumor was diagnosed as the metastasis from lung cancer based on the immunohistochemistry findings and the concordance of the somatic mutations.

### Patient 2

A 47-year-old woman was diagnosed with cStage IIIB (T4a N2 M0) gastric cancer according to the Japanese Classification of Gastric Carcinoma, 3rd English edition [11].

We performed distal gastrectomy, D2 lymph node dissection, dissection of the lymph nodes around the hepatoduodenal ligament, and cholecystectomy. We also resected the bilateral ovaries, because enlargement of the ovaries was detected during the operation. A histopathological examination revealed pStage IV (T4a N2 M1) gastric cancer. Peritoneal lavage cytology was negative for cancer. The enlarged ovaries were diagnosed as metastasis from gastric cancer.

After gastrectomy, the patient underwent adjuvant chemotherapy with S-1 for one year. A colon tumor was detected by a detailed examination after a fecal occult blood test, which was performed for screening purposes 6 years after gastrectomy.

Endoscopically, the colon lesion was a 40 mm submucosal tumor-like elevated tumor. A histopathological examination of the biopsy specimen showed poorly differentiated adenocarcinoma and signet ring cell carcinoma. No other tumor or metastatic lesion was detected by CT or FDG-PET. Although the colon tumor was suspected to be metastasis from gastric cancer, the possibility of primary colorectal cancer could not be completely ruled out. If the tumor was colon cancer, the clinical stage was IIA (T3 N0 M0) according to the Union International for Cancer Control-TNM, 8th edition.

**Fig. 4:**
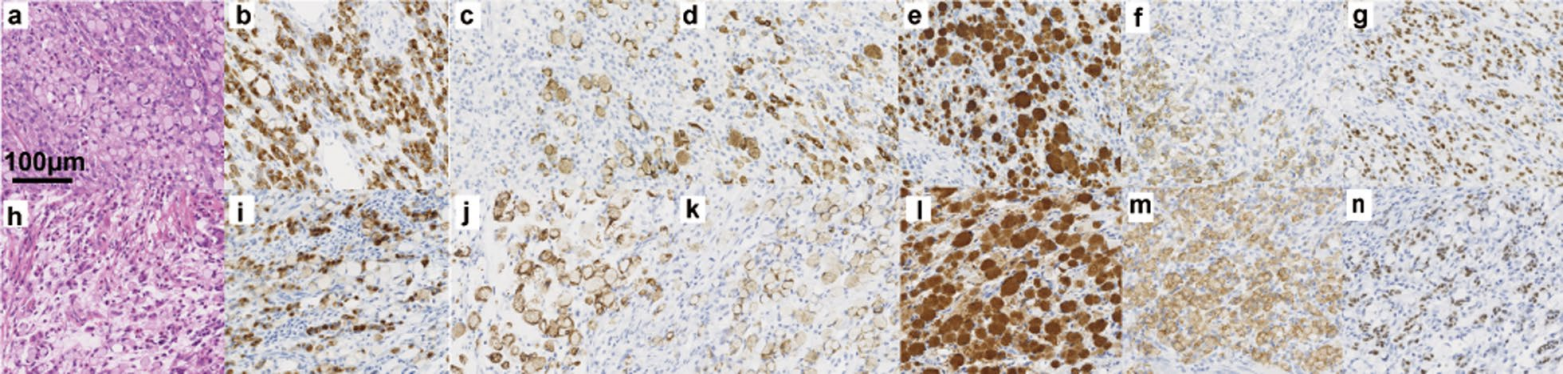
Histopathological findings of the colon tumor (**a**–**g**) and previously resected gastric cancer (**h**–**n**). The colon tumor consisted of signet ring cell carcinoma that mainly existed in the submucosa (**a**). The tumor cells were similar to the previously resected gastric cancer (hematoxylin–eosin staining) (**h**). The tumor cells of the colon tumor were positive for CD10 (**b**), focally positive for MUC2 (**c**), positive for MUC5AC (**d**), HGMucin (**e**), focally positive for MUC6 (**f**), and positive for CDX2 (**g**). The tumor cells of the gastric tumor were positive for CD10 (**i**), focally positive for MUC2 (**j**), MUC5AC (**k**), HGMucin (**l**), MUC6 (**m**), and CDX2 (**n**)

The patient underwent laparoscopic sigmoidectomy with D2 lymph node dissection. The patient participated in “Project HOPE” and WES and GEP were conducted [12, 13]. Macroscopically, the lesion was a 27-mm submucosal-like nodule. Microscopically, the tumor was signet ring cell carcinoma (Fig. 4a). No mucosal lesion was observed, and the tumor cells existed mainly in the submucosa. Immunohistochemical staining of the sigmoid colon tumor and previously resected gastric cancer were performed (Fig. 4b–g, i–n). After matches were made according to the histological type and the mucin phenotype, the colon tumor was thus suspected to have been metastasis of gastric cancer. However, the possibility of primary colorectal cancer could not be completely ruled out because the mucin phenotype of both the colon tumor and gastric cancer was a gastrointestinal type, not a gastric type. The WES results showed that the colon tumor had no specific mutational signature (data not shown).

To confirm that the origin of colon tumor, we conducted a somatic mutation comparison of the two tumors, as we did with Patient 1. DNA was extracted from formalin-fixed paraffin-embedded tissues resected in gastrectomy and sigmoidectomy. Panel sequencing revealed 11 and 11 mutations in the gastric and colon tumors, respectively (Additional file 2: Table S1). None of them were driver mutations. Of the 11 mutations in the gastric tumor, 4 completely matched those of the colon tumor.

Based on the results of immunohistochemical staining and comparison of the somatic mutations, the colon tumor was diagnosed as metastasis from gastric cancer.

## Discussion

We encountered two patients in whom NGS was helpful for making a differential diagnosis of primary and metastatic tumors. From the mutation comparisons, one patient was diagnosed with stomach metastasis from lung cancer, while the other was diagnosed with colonic metastasis from gastric cancer.

Metastatic gastric tumors are rare; the incidence was reported to be 0.2–5.4% among all gastric tumors [17–19]. Among sites of lung cancer metastasis in the gastrointestinal tract, the small intestine and colorectum are common metastatic sites, while gastric metastasis is rare [20]. The dominant histopathological type of gastrointestinal metastasis from lung cancer is reported to be squamous cell carcinoma; however, the histopathological type of Patient 1 was adenocarcinoma [21, 22]. In a differential diagnosis using immunohistochemical staining, TTF-1 is a specific marker of the lung and thyroid gland and is also used to identify the primary lesion of adenocarcinoma of unknown primary. Although a metastatic gastric tumor could have been highly suspected if TTF-1 had been evaluated at the time of biopsy, there are reports describing TTF-1-positive gastric cancer, colorectal cancer, or schwannoma [15, 16, 23, 24]. Thus, TTF-1-positive primary gastric cancer could not be completely ruled out. There is no specific immunohistochemical marker for the diagnosis of gastric cancer. In Patient 1, intratumoral heterogeneity or heterogeneity between the primary and metastatic lesions (the lung biopsy specimen was negative for TTF-1 while the mediastinal lymph node biopsy was positive for TTF-1) made the differential diagnosis more difficult. Moreover, the gastric tumor was macroscopically a type 2 tumor, and the histopathological findings were different from those of the lung biopsy specimen.

One of the points used for differentiation between a metastatic tumor of the gastrointestinal tract and a primary tumor is the macroscopic type. Another possible method for the differential diagnosis is tissue-specific immunohistochemical staining. Metastatic tumors should always be kept in mind when multiple tumors are found, and it is necessary to perform immunohistochemical staining of biopsy specimens to avoid unnecessary surgery.

From stomach cancer, liver, lung, and bone metastases are the most common types of hematogenous metastasis [25]. Colorectal metastasis from gastric cancer was reported in rare cases, and is difficult to distinguish from poorly differentiated colorectal cancer [26–28].

We previously compared the somatic mutation concordance of metastatic and multiple cancer [9]. In that study, a whole exome analysis involving approximately 20,000 genes was performed and it was found that a complete mutation match (mutation site and mutation pattern on the chromosome) of 5 or more genes was extremely rare (0.013%) in multiple cancers, regardless of the tumor mutation burden. Additionally, the number and rate of somatic mutation concordance did not differ substantially between the synonymous and nonsynonymous mutations described in that study. In this study, 28 gene mutations were completely matched in Patient 1, and two of the 28 matched gene mutations were driver mutations. Although only 4 mutations matched in Patient 2, the probability of a complete match of 4 genes in multiple cancers (primary gastric and colon cancer) appeared to be extremely low, considering that in this study we used a panel test involving 409 genes, which is approximately 1/50 of the previously reported gene mutation comparison. Using a cancer-specific gene panel sequencing enables an inexpensive and efficient mutation comparison for the different diagnosis of a metastatic tumor in comparison to a comprehensive mutational analysis, which would appear to contribute to its clinical application.

Recently, with the advancement of NGS, many studies have reported the comparison analysis of gene alterations and the gene expression of paired primary and metastatic lesions, and attempted to identify the primary lesion based on the molecular profiles [4–6, 8–10, 29, 30]. Paired primary and metastatic lesions have been reported to show a similar mutational landscape and mutational signature [4, 30, 31]. However, this is the first report to demonstrate that the results of a mutational analysis using NGS were useful for the diagnosis of metastatic cancer. A gene mutation signature analysis was helpful for identifying the primary organ of the tumor in this study.

## Conclusion

We described two patients with multiple tumors in which a somatic mutation comparison using NGS was useful for the diagnosis of a metastatic tumor.

## Supplementary Information

**Additional file 1: **Immunohistochemical staining of thyroid transcription factor-1 (TTF-1) in the gastric tumor biopsy and gastrectomy specimens.

**Additional file 2: **Somatic mutations identified in panel sequencing.

## Data Availability

After de-identification, individual participant data that underlie the results will be shared with the investigators when the proposed use of the data is approved by the investigators from the Shizuoka Cancer Center identified for this purpose. Proposals should be directed to m.terashima@scchr.jp.

## Abbreviations

NGS: Next-generation sequencing
CEA: Carcinoembryonic antigen
CA19-9: Cytokeratin 19 fragment
CT: Computed tomography
FDG-PET: ^18^F-fluorodeoxyglucose-positron emission tomography
CK: Cytokeratin
TTF-1: Thyroid transcription factor-1
WES: Whole exome sequencing
GEP: Gene expression profiling
