# Supplementary material for: Report of two patients in whom comparisons of the somatic mutation profile were useful for the diagnosis of metastatic tumors
Source: Surg Case Rep. 2022 Dec 2;8:214. doi: 10.1186/s40792-022-01566-8 (PMC9718898; doi:10.1186/s40792-022-01566-8)
Supplement: Supplementary file 1 — Additional file 1: Immunohistochemical staining of thyroid transcription factor-1 (TTF-1) in the gastric tumor biopsy and gastrectomy specimens. [file 40792_2022_1566_MOESM1_ESM.pdf]

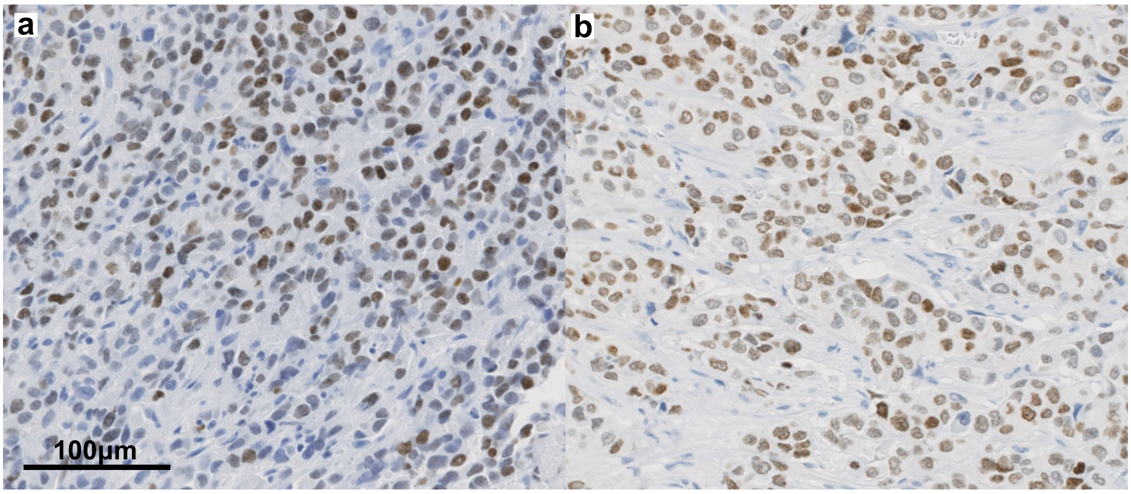

Figure S1: Immunohistochemical staining of thyroid transcription factor-1 (TTF-1) in the gastric tumor biopsy and gastrectomy specimens

The results were obtained after performing a mutational signature analysis of the gastric tumor. The tumor cells of the gastric tumor biopsy (a) and gastrectomy (b) specimens were positive for TTF-1.
