# Supplementary material for: Report of two patients in whom comparisons of the somatic mutation profile were useful for the diagnosis of metastatic tumors
Source: Surg Case Rep. 2022 Dec 2;8:214. doi: 10.1186/s40792-022-01566-8 (PMC9718898; doi:10.1186/s40792-022-01566-8)
Supplement: Supplementary file 2 — Additional file 2: Somatic mutations identified in panel sequencing. [file 40792_2022_1566_MOESM2_ESM.pdf]

Table S1. Somatic mutations identified in panel sequencing

Patient	Gene mutation		Driver	Organ
Patient 1	*ADAMTS20*	c.1077G>T	missense	L
		c.1503C>T		L
		c.310T>C	missense	S
		c.3463C>A	missense	common
		c.3938G>A	stop gained	L
	ADGRL3	c.2477A>T	missense	S
		c.2515A>G	missense	L
		c.3116A>T	missense	L
		c.3498T>A	missense	S
	AFF3	c.2754C>T		L
	AKAP9	c.115A>G	missense	S
		c.8646+8G>T		S
	ALK	c.1312C>A	missense	common
	ARID2	c.2791C>T	driver	S
	BCL11A	c.980C>A	missense	S
	BCL11B	c.*22G>T		S
		c.2184C>A		S
	BIVM-ERCC5	c.3077A>T	missense	L
	CDH11	c.2290G>T	missense	S
		c.2335T>A	missense	L
	CDH2	c.1305C>A		S
		c.1494C>A		L
	CDH20	c.2218C>G	missense	S
	CHEK1	c.1342G>T	driver	S
	CREBBP	c.4416G>T	missense	L
	CSMD3	c.10373C>A	missense	S
		c.1325C>A	missense	S
		c.4541C>A	missense	S
		c.50G>T	missense	L
		c.7697-1G>T	splice acceptor	L
	CTNNA1	c.2480A>G	missense	L
	DDR2	c.624C>T		common
	DEK	c.944delA	frameshift	S
	DICER1	c.382G>A	missense	L
	DPYD	c.1201G>T	missense	common
	DST	c.10683G>T		common
		c.10776G>C		L
		c.14353G>C	missense	common
	EGFR	c.3028G>C	missense	L
	EP400	c.1536C>T		L
	EPHA3	c.191C>A	missense	L
		c.2311C>A	missense	L
	EPHB1	c.1882+6G>T		L
		c.2278G>T	missense	L
		c.532C>A	missense	L
	EPHB6	c.301C>A	missense	L
	ERBB4	c.2824C>T	missense	L
		c.3818G>T	missense	L
		c.557-3C>A		L
		c.726G>A		L
	ERCC2	c.453C>T		S
		c.760C>T	missense	L
	ERCC5	c.1715A>T	missense	L
	ETS1	c.924G>A		S
	FBXW7	c.173A>T	missense	L
	FGFR2	c.2180A>G	missense	S
		c.696A>T		S
	FLT4	c.667C>A	missense	common
	FOXL2	c.655C>T	driver	common
	GATA3	c.-40delG		common
	GRM8	c.57C>A		L
	GUCY1A2	c.2184G>T		L
	HIF1A	c.317_319delATG	deletion	common
	HNF1A	c.1124_1125delGC	driver	S
	HOOK3	c.1406G>T	missense	L
	IL6ST	c.2137C>T		L
	IL7R	c.1124G>A	missense	common
	ITGA9	c.1141+1G>T	splice donor	S
	JAK2	c.1531G>T	missense	L
	JAK3	c.2805+4C>G		L
	KEAP1	c.1075C>T	driver	S
	KLF6	c.-32G>A		L
	KMT2D	c.13300G>A	missense	S
	LRP1B	c.11024G>T	missense	S
		c.11416G>A	missense	L
		c.11973delG	frameshift	common
		c.3352G>A	missense	L
		c.8609A>T	missense	L
		c.8936G>T	missense	common
	MAGEA1	c.622C>A	missense	L
	MAGI1	c.2587G>A	missense	S
	MARK1	c.1011C>T		L
	MCL1	c.559C>T	missense	S
		c.658G>T	missense	S
	MET	c.690G>C		L
	MLLT10	c.2112C>T		S
	MN1	c.723G>T		S
	MTOR	c.5347G>T	missense	L
		c.7411A>T	missense	common
	MTR	c.3317G>T	missense	common
	NCOA2	c.905G>T	missense	L
	NDE1	c.*612C>T		L
	NLRP1	c.2205G>T	missense	common

Patient	Gene mutation		Driver	Organ
Patient 2	*ALK*	c.2535T>C		S
	CIC	c.87T>G		C
	CTNNA1	c.1351C>T	stop gained	common
	ERBB4	c.884-8_884-7delTT		S
	FH	c.693T>C		C
	GATA3	c.-40delG		common
	IGF2	c.518delC	frameshift	C
	IL7R	c.399T>C		C
	KNL1	c.829G>T	missense	C
	MYB	c.1697C>T	missense	S
	MYH9	c.3217G>T	stop gained	S
	NOTCH4	c.36_47delGCTGCTGCTGCT	deletion	common
	NUP214	c.1099G>A	missense	common
	PDE4DIP	c.1679G>A	stop gained	S
	PTCH1	c.318C>T		S
	SYNE1	c.12180G>T	missense	S
		c.8508A>C	missense	C
	TET2	c.1415C>T	missense	C

NOTCH4	c.1942A>T	missense	S
	c.311C>T	missense	L
	c.449C>A	missense	common
NTRK1	c.1873G>T	missense	common
NTRK3	c.164C>A	missense	S
NUP214	c.4092G>C	missense	S
NUP98	c.609T>A		L
PAX5	c.212+6G>T		S
PAX8	c.533C>A	missense	L
	c.912C>A		S
PDE4DIP	c.6954A>G		S
PDGFRA	c.1460G>T	missense	S
PDGFRB	c.748C>T	missense	S
PGAP3	c.387G>T		S
PIK3C2B	c.3249+4A>G		S
PIK3CG	c.1075C>A	missense	common
PIK3R1	c.1963G>T	missense	common
PKHD1	c.4838G>T	missense	S
PML	c.464A>T	missense	L
PPARG	c.764C>T	missense	S
PTCH1	c.3768G>T		S
PTPRD	c.1262C>G	missense	S
	c.136C>A	missense	L
	c.1959delT	driver	L
	c.3189G>T		L
PTPRT	c.1102G>T	missense	L
	c.3988C>T	missense	L
RALGDS	c.2515G>T	stop gained	S
RB1	c.1651G>T	driver	S
	c.1733A>T	missense	L
RET	c.2373T>C		S
RNF213	c.13756A>C	missense	S
RUNX1	c.138C>A	missense	S
RUNX1T1	c.1248G>T	missense	L
	c.1745C>A	missense	L
	c.-305G>T		S
SAMD9	c.2680A>G	missense	L
SF3B1	c.1719+1G>T	splice donor	common
	c.596G>T	missense	L
SMARCA4	c.2338G>T	driver	L
SMO	c.849C>A		common
SOX11	c.1000G>C	missense	L
	c.47G>T	missense	L
STK11	c.667G>A	missense	L
SUFU	c.1449A>G		S
SYNE1	c.7768G>T	missense	S
TBX22	c.1150C>A	missense	S
TCL1A	c.121-5C>A		common
TGFBR2	c.1181G>C	missense	common
TLR4	c.1037C>A	missense	common
	c.1573C>G	missense	L
	c.1623delG	frameshift	S
	c.162C>A	missense	S
	c.1639C>A	missense	S
	c.2015A>G	missense	S
	c.423A>G		L
TNK2	c.730G>T	stop gained	L
TP53	c.783-1G>T	driver	common
TRIP11	c.2317A>T	missense	common
WHSC1	c.3771G>A		L
WT1	c.922C>A	missense	common
ZNF521	c.3406C>A	missense	L

S, stomach (gastrectomy); L, lung (biopsy); C, colon (colectomy); common, mutation detected in both organs.
